# Supplementary material for: Causal interplay between lactose intolerance and gut microbiota: a combined bidirectional Mendelian randomization and in vivo validation study
Source: Front Nutr. 2026 Jun 1;13:1803337. doi: 10.3389/fnut.2026.1803337 (PMC13265576; doi:10.3389/fnut.2026.1803337)
Supplement: Supplementary file 1 [file Data_Sheet_1.zip › supplementary materials/Forward/leave one out cross validation/ebi-a-GCST90027608.finngen_R12_E4_LACTONAS.pdf]

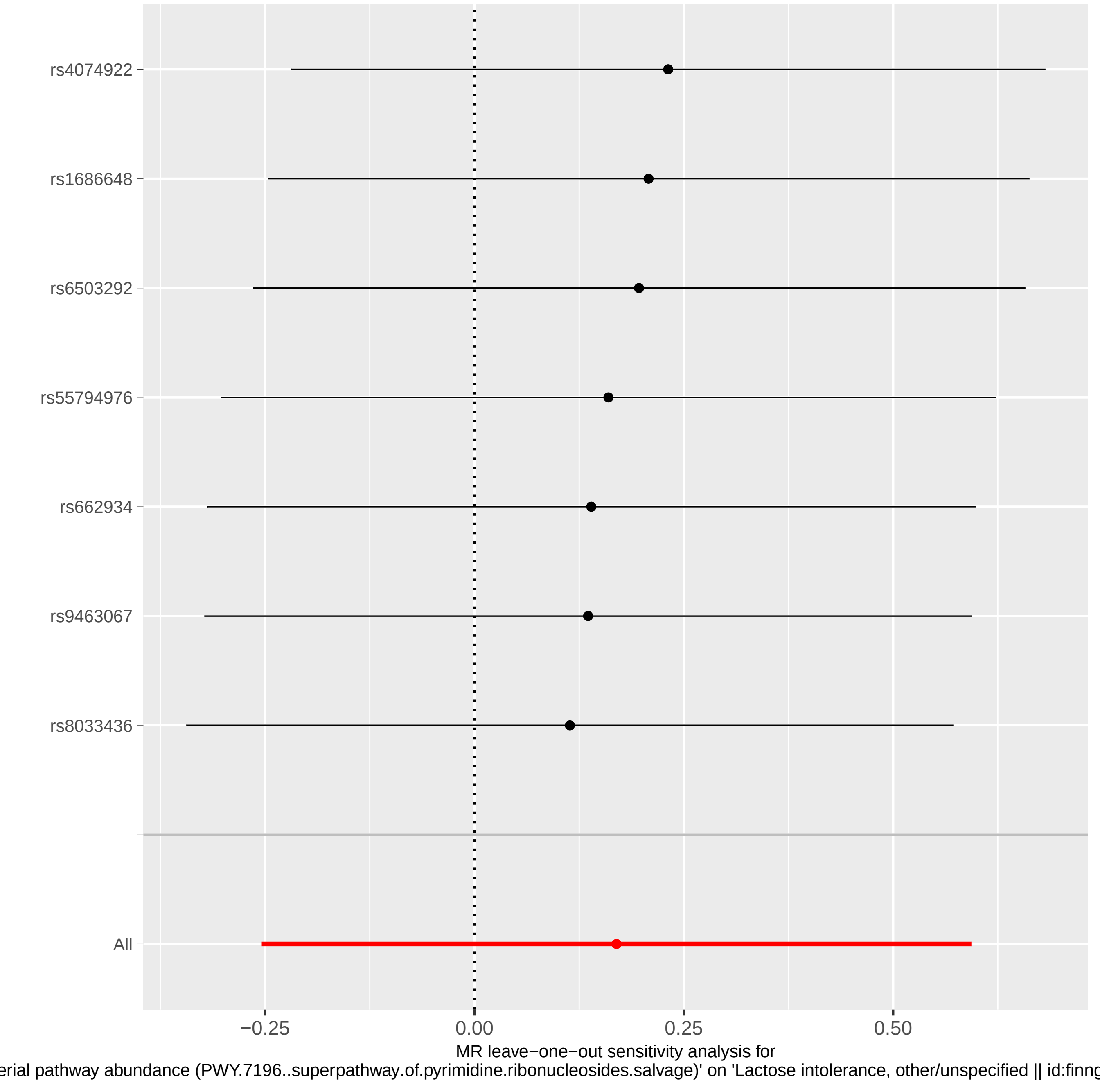

rs4074922
rs1686648
rs6503292
rs55794976
rs662934
rs9463067
rs8033436
All
−0.25
0.00
0.25
0.50
MR leave−one−out sensitivity analysis for
erial pathway abundance (PWY.7196..superpathway.of.pyrimidine.ribonucleosides.salvage)' on 'Lactose intolerance, other/unspecified || id:finng
